# Supplementary material for: Asynchronous domain dynamics and equilibration in layered oxide battery cathode
Source: Nat Commun. 2023 Dec 18;14:8394. doi: 10.1038/s41467-023-44222-x (PMC10728132; doi:10.1038/s41467-023-44222-x)
Supplement: Supplementary file 1 — Supplementary Information [file 41467_2023_44222_MOESM1_ESM.pdf]

Supplementary Materials for

Asynchronous Domain Dynamics and Equilibration in Layered Oxide Battery Cathode

Zhichen Xue[1,2], Nikhil Sharma[3], Feixiang Wu[1, *], Piero Pianetta[2], Feng Lin[4], Luxi Li[5, *], Kejie Zhao[3,*], Yijin Liu[6*]

1.School of Metallurgy and Environment, Central South University, Changsha, 410083, China
2.Stanford Synchrotron Radiation Lightsource, SLAC National Accelerator Laboratory, Menlo Park, California 94025, USA
3.School of Mechanical Engineering, Purdue University, West Lafayette, IN, 47906 USA
4.Department of Chemistry, Virginia Tech, Blacksburg, VA, USA
5.X-ray Science Division, Argonne National Laboratory, Lemont, IL, 60439, USA
6.Walker Department of Mechanical Engineering, The University of Texas at Austin, Austin, Texas 78712, USA

Supplementary Text

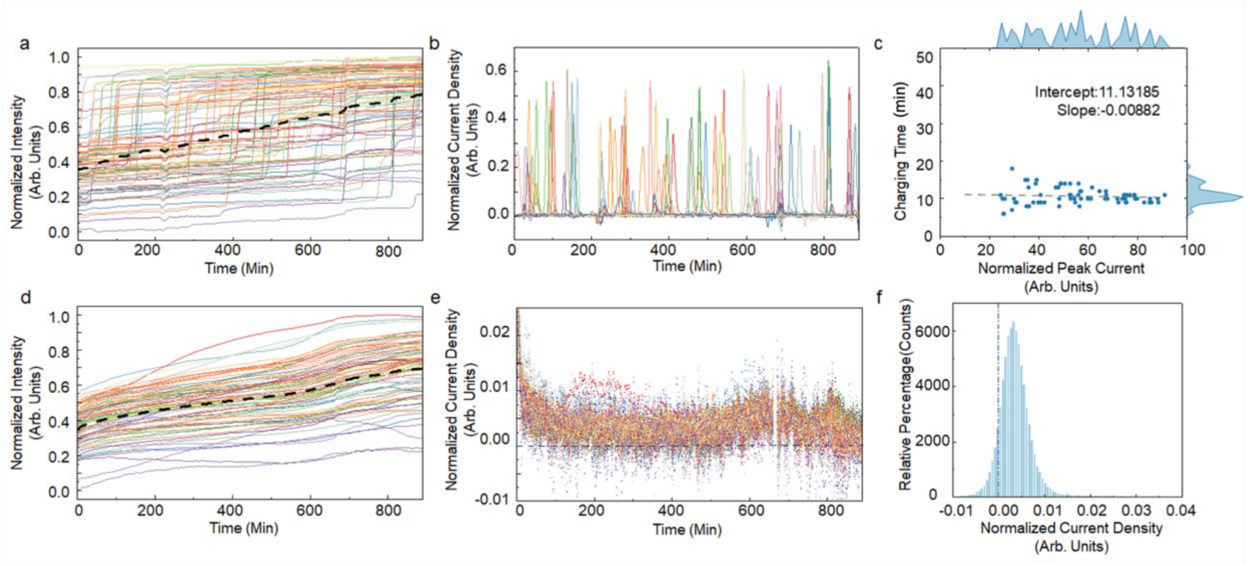

Figure S1. Dynamic behaviors of nearly 100 particles during the first (**a** to **c**) and the second charge (**d** to **f**). Evolution in the average particle intensity during the first and second charge (**a** and **d**), and the evolution of the local current intensity over the particles (**b** and **e**), and their respective statistics (**c** and **f**).

Although the asynchronous to synchronous transformation of the particle behaviors have been reported [1], here we provide a more detailed statistical analysis. With the nearly 100 particles identified and segmented, we plot their respective SOC and local current density as a function of time during the first charge and the second charge. The first-charge asynchronous activity of the particles results in distinct charging pulses in Figure S1b. In contrast, Figure S1e shows a more slowly progressive charging process during the second charge. In the first charge, it appears that each charging pulse lasts around 10 minutes, regardless of the particle size and shape. In the second charge, we observe that majority of the particle are seeing a charging current throughout the charging process and their SOC would increase monotonically. However, there are a small number of particles that could experience discharging current at some point during the second charge. This is a topic of great interest but is beyond the scope of this work.

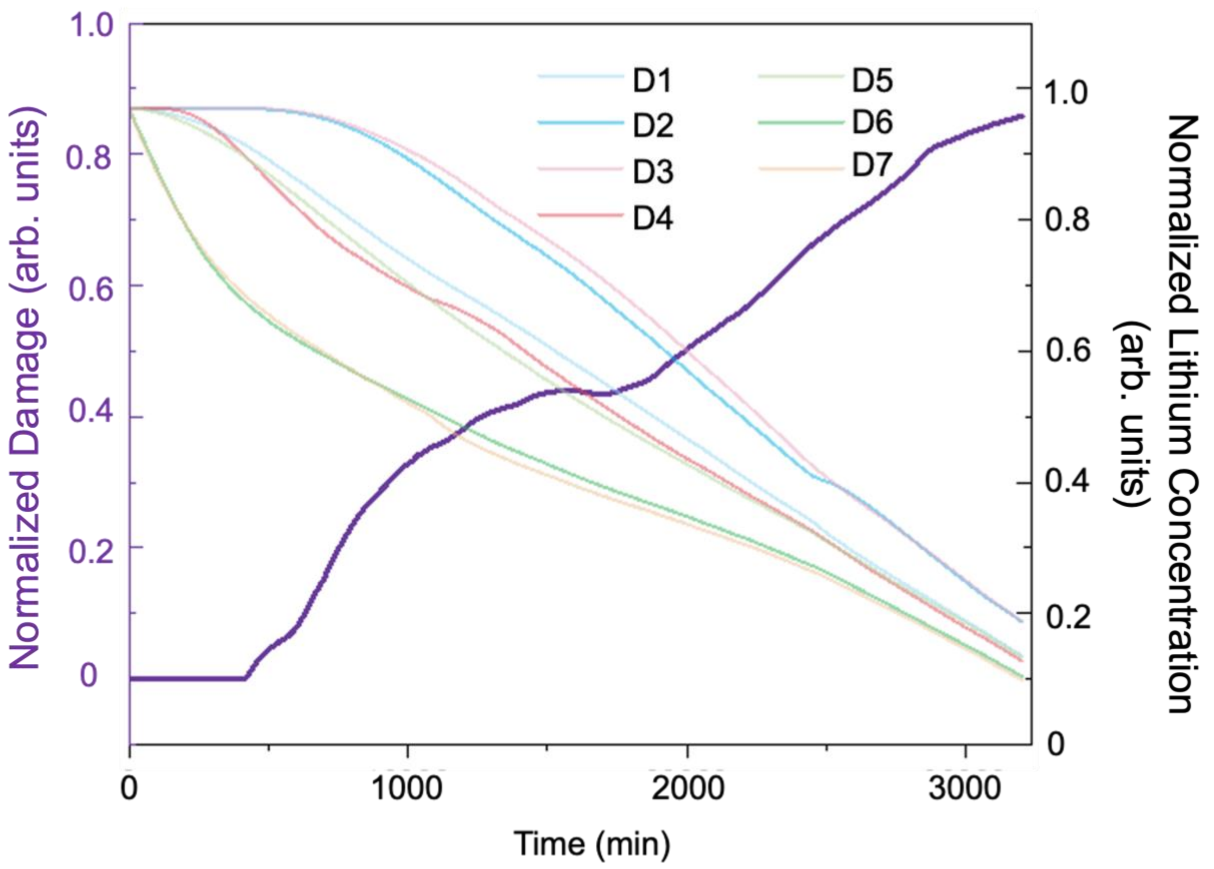

Figure S2. FEM modeling results on the normalized damage for the whole particle (left) and lithium concentration profiles for the sub-particle domains evolve (right) over time during the charging process.

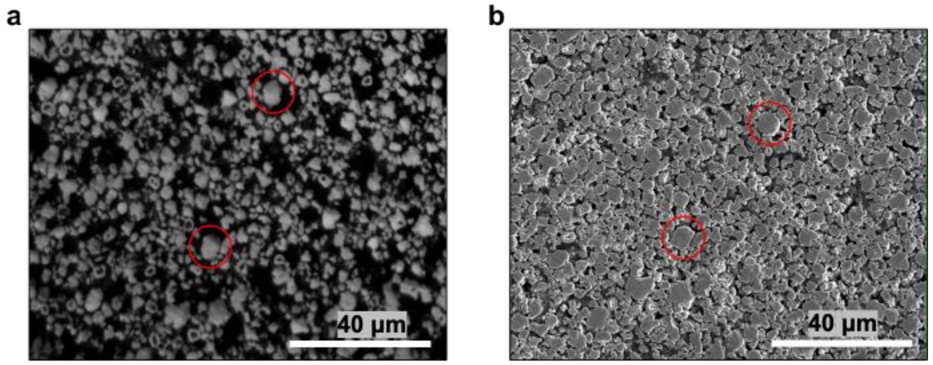

Figure S3. Optical image (**a**) of the polished NMC cathode with corresponding SEM image **(b)**.

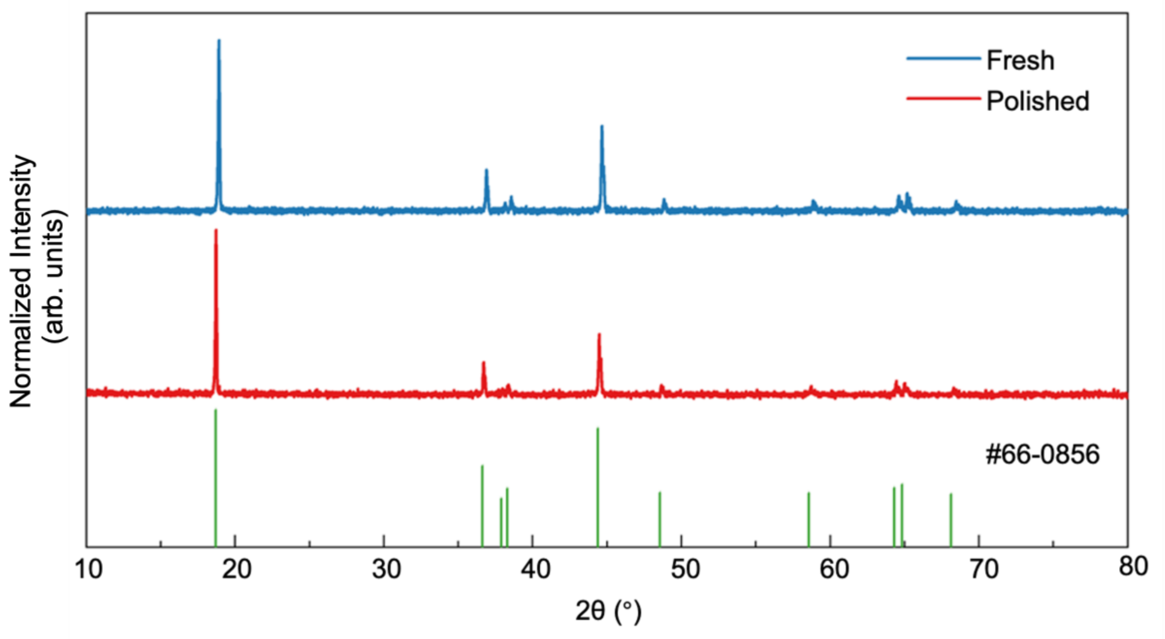

Figure S4. The XRD patterns of fresh and polished NMC cathode electrode. The crystal structures of both original and polished electrodes match the reference crystal structure with the R-3m space group (#66-0856). The XRD peaks (Figure S4) observed in both patterns align with the expected positions and intensities for NMC, indicating that the crystal structure remains unchanged after the polishing treatment. These results provide evidence that the polishing process does not alter the crystal structure of the NMC electrodes.

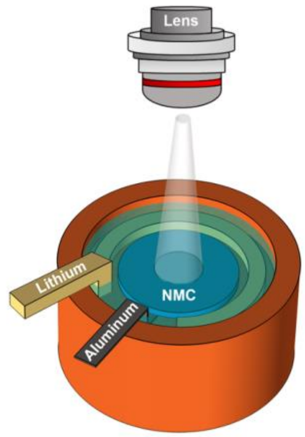

Figure S5. Schematic illustration of the experimental setup designed for operando optical imaging of battery electrodes during controlled electrochemical cycling in an inert gas environment. The cell comprises a polished NMC cathode (working electrode, W.E.) surrounded by a Li metal ribbon (counter electrode, C.E.) within a liquid electrolyte container that is connected to a potentiostat.

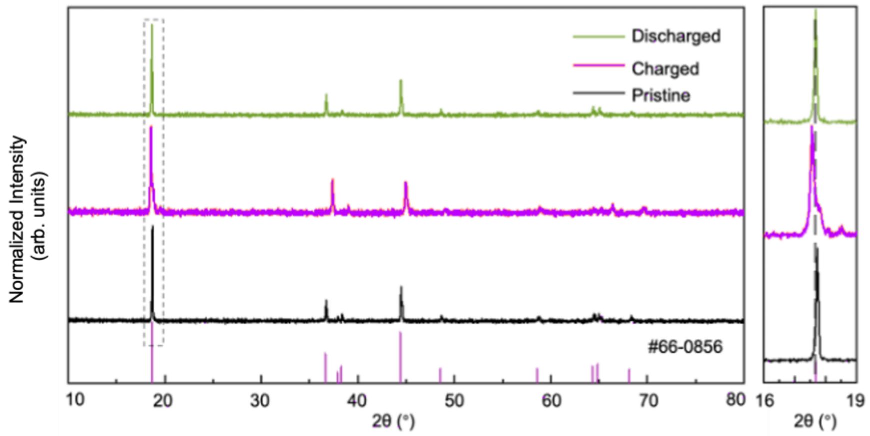

Figure S6. Ex-situ XRD pattern on thin film electrodes that were harvested at different electrochemical states. All the peaks in the XRD patterns match well with the reference pattern #66-0856, indicating a well-formed layered structure. The results confirm that the expected electrochemical reactions did occur in our cells. Notably, at high cell SOC, a significant and thermodynamically forbidden phenomenon was observed: phase separation, as indicated by a pronounced shoulder at the (003) XRD peak. This pseudo phase separation was attributed to autocatalytic behaviors between different particles in the electrode. Subsequently, after the discharge, the peak splitting phenomenon disappears, with the (003) peak returning to its original position and shape.

Data processing and Finite Element Modeling (FEM)

1.Image Analysis Methodology

1.1 Image Stack Registration

The video recorded using the optical microscope was first converted from RGB color format into gray intensity. To correct for the image jittering, which is caused by the instability of the sample stage and/or the lens, all the images underwent a rigid image registration step using the ImageJ software. Note that, although the image brightness and contrast changes as a function of the SOC, the image registration process is very robust and precise. This is very similar to the image registration step in X-ray spectro-microscopy. Several different algorithms have been demonstrated to be effective for this task.

1.2 Particle Segmentation

To investigate the dynamic behaviors of different particles, all the particles imaged in our field of view were segmented using Avizo software (Figure S7). As shown in Figure S7, nearly 100 particles were identified in this process and were subjected to the downstream analysis. A high throughput particle quantification approach was developed to survey all these particles to evaluate their similarity and differences (see section 1.4 below for more details).

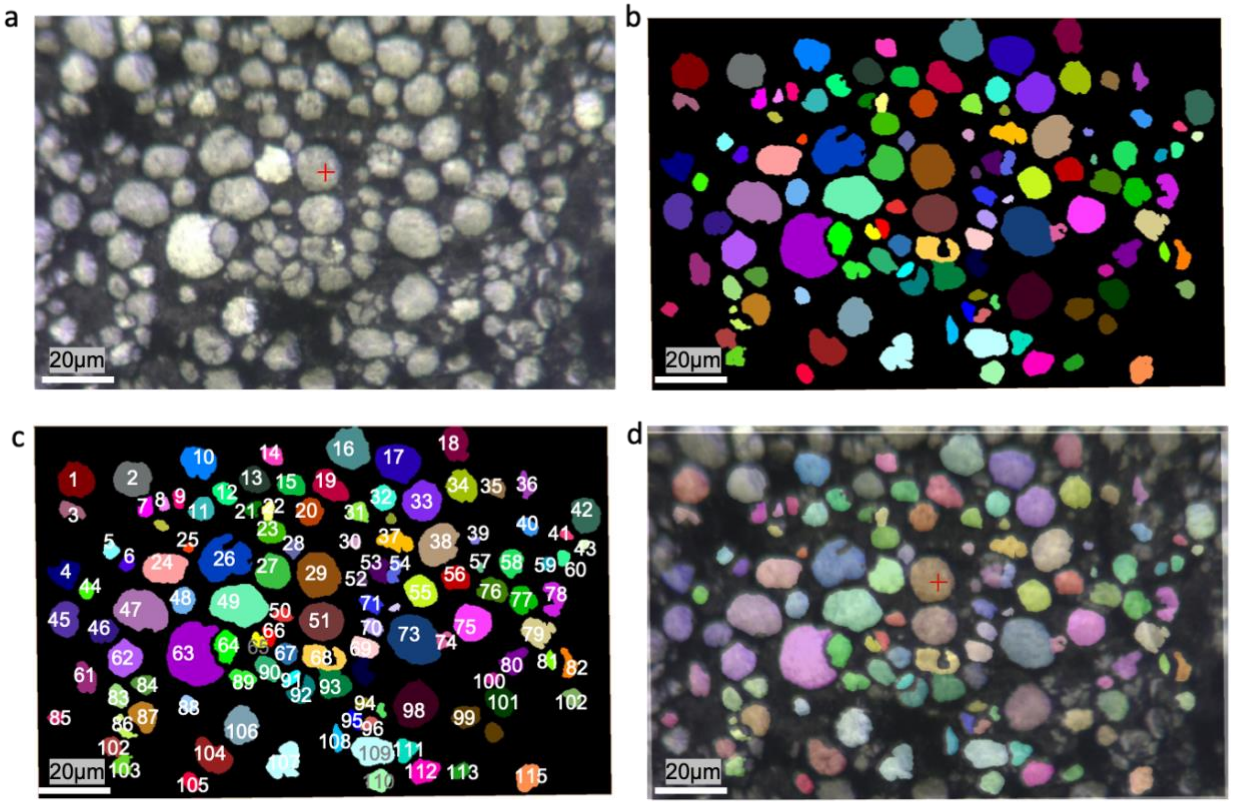

Figure S7. (A) Operando optical images of the electrode (scale bar 20 μm). The segmented image **(B)** and label **(C)** showing nearly 100 particles. **(D)** Overlay of the segmentation result and the original image.

1.3 Calibration of Intensity and SOC

In this study, we establish a quantitative correlation between NMC electrode's optical density and its SOC. While the particle-to-particle variation is a dominant feature in the firs charge, as we have reported, the particles behave in a more synchronized fashion in the later cycles. Therefore, it provides an opportunity for us to correlate the optical density with the SOC. More specifically, through the analysis of image intensity changes during the second charge and discharge cycle, it becomes evident that the overall intensity is monotonically correlated with the SOC. The segmented nearly 100 particles also allow us to evaluate this correlation with statistical significance (Figure S8). This nicely echoes with the reports in the literature that changes in the SOC of the electrode material will lead to changes in the dielectric constant, which in turn will cause changes in the optical intensity measured with an optical microscope [3, 4, 5].

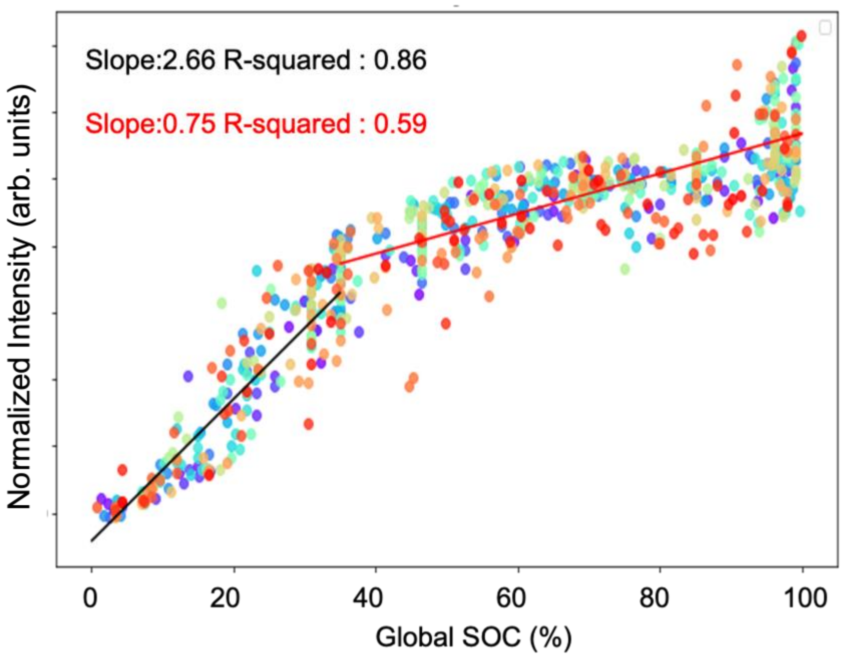

Figure S8. Linear regression plot illustrating the relationship between particles' optical density and the SOC during the second charge. Each color represents a unique particle. The linear regression reveals a positive correlation with a slope of 2.66 and 0.75 with R-squared value of 0.86 and 0.59 respectively.

1.4 High-throughput Particle Analysis and Clustering

With nearly 100 particles identified and segmented, we developed a streamlined workflow to survey all the particles and to determine their similarities and differences. A schematic of our workflow is shown in Figure S9. We start with the registered image stack and label segmentation results to extract and scrutinize the particles one at a time. To group the particles with similar behavior, we first extract the averaged chemical state, intraparticle heterogeneity, and dynamic intensity changes of each particle. We evaluate the averaged characteristics for all the particles and then identify the ones with most distinct differences in different aspects. This process is done in an iterative manner and abnormal particles are singled out for further analysis. During this process, we found some particles with relatively insignificant changes in their intensity, which may indicate that they are less electrochemically active and are not subjected to further analysis. This approach facilitates the screening and clustering of particles, pinpointing those warranting detailed study based on their electrochemical behavior and other attributes. One abnormal particle was singled out for in-depth analysis in the main text (Figure 2).

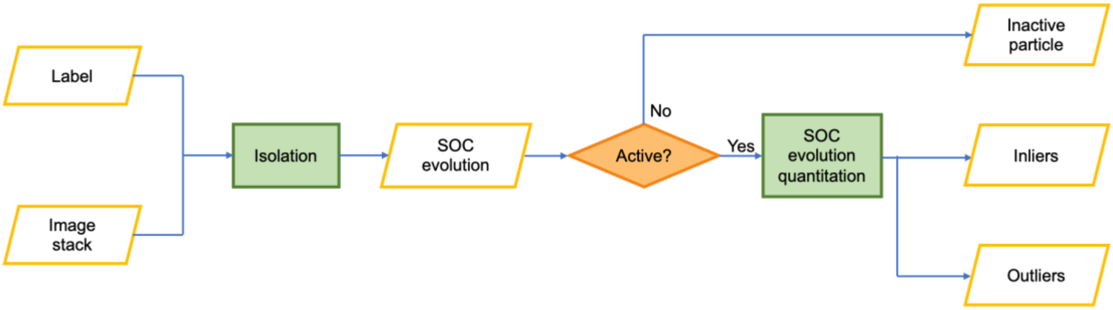

Figure S9. Workflow for analyzing and classification of the individual particle's SOC evolution.

To determine the average intensity evolution of a certain particle, the label image (Figure S10 C) and the stack of the previously registered optical photos are used as input. The average SOC of the particle (Figure S10a) is obtained by averaging the intensity of all the pixels over the corresponding particle. The derivative of this curve can be interpreted to be the local current evolution over the particle (Figure S10b).

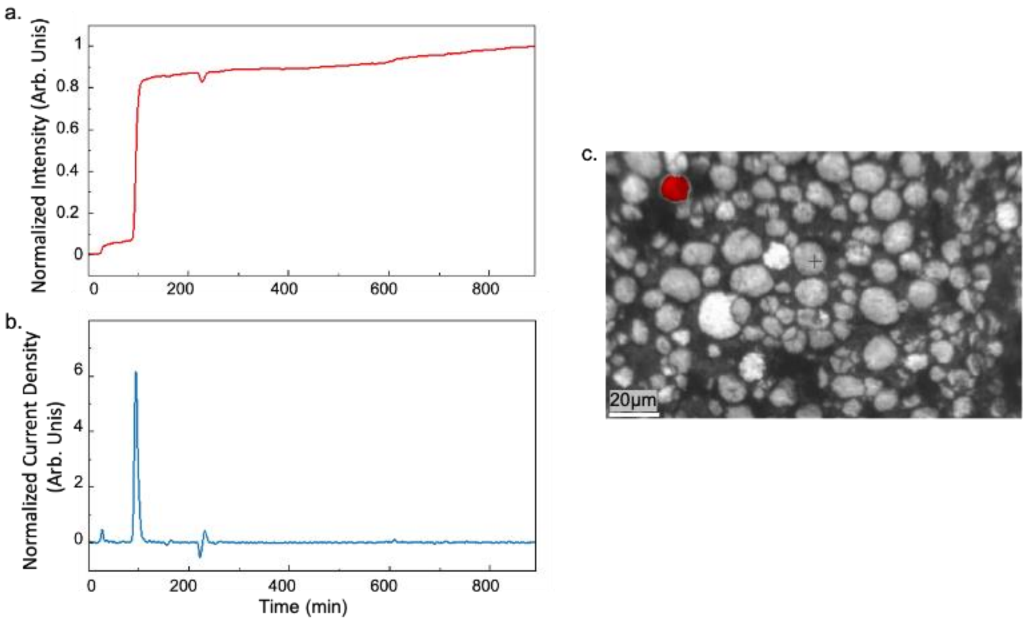

Figure S10. The analysis of a randomly selected particle in the field of view. (**A**) the particle's averaged optical density evolution during charging, (**B**) normalized current density over the same particle and (**C**) particle label and morphology.

1.5 Electrochemical Activity Maps

As we have established a positive correlation between the optical density and the SOC, we can utilize optical images acquired at different time to evaluate the local electrochemical activities. Normalized electrochemical activity maps (current density map, at each time t) were obtained by dividing the pixel values of frame pairs (separated by a time interval, Δt) as follows：

$$\text{Electrochemical actively map(t)} = \frac{\frac{\text{Frame(t)}}{\text{Frame(t}-\Delta\text{t)}}-1}{\Delta t} \qquad (1)$$

The differential contrast obtained shows the fractional variation in intensity between the two frames, with the contrast centered at 0. This method eliminates constant or slowly changing background effects and inconsistencies in sample illumination, which allows for the highlighting of changes relevant to the electrochemical activity. Negative pixel values indicate a decrease in optical density (discharging), while positive values indicate an increase in optical density (charging).

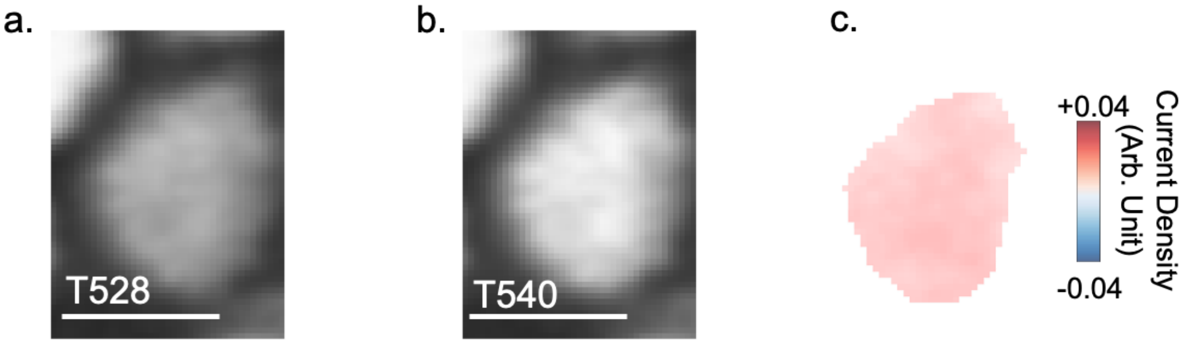

Figure S11. An example to illustrate the calculation of electrochemical activity map over an NMC particle. (**A**) frame (t, t=540) of particle 13#, (**B**) frame (t-△t, △t=12) of particle 77#, (**C**) the calculated electrochemical activity map. Scalebars in panels are 3 μm.

1.6 Circular plot of the local current distribution

To characterize the anisotropy of the local current over the particle at different times, the above-described electrochemical activity map is converted into polar coordinates. Starting at the particle's center of weight, we divided it into many fan-shape segments and calculate the averaged value and standard deviation as a function of the angle. The 2D chemical activity map is effectively projected onto the angular space, which allows us to visualize the sequential activity of different domains. In these circular plots, a large value in a certain direction means that the higher electrochemical activity of the regions along this direction, and a value close to 0 means that the activity of the pixels in this direction is low.

2. FEM

We perform multiphysics-based finite element analysis to understand intra-particle charging behavior. The 2D model geometry (Figure S12) consists of a current collector at the bottom and a carbon binder matrix (a uniform 50% porous) partially covering the NMC active particle (10 μm diameter). The liquid electrolyte fills the rest of the space, and Li-ion flows within the liquid electrolyte to the Lithium anode at the top of the diagram upon charging. Electrons travel only through the carbon binder to the current collector at the bottom. Mechanics equations to compute strain and stress in domain bulk and at the boundaries are given below:

Deformation kinematics:

$$\boldsymbol{\varepsilon} = \frac{1}{2}\left((\nabla u)^{\mathrm{T}} + (\nabla u)\right) \qquad (2)$$

$$\boldsymbol{\varepsilon}_{\mathbf{Li}} = \frac{1}{3}\Omega\left(c_s - c_{s_{ini}}\right)\mathbf{I} \qquad (3)$$

$$\boldsymbol{\varepsilon} = \boldsymbol{\varepsilon}_{\mathbf{el}} + \boldsymbol{\varepsilon}_{\mathbf{Li}} \qquad (4)$$

Where $\boldsymbol{\varepsilon}$ is the total strain, u is the displacement, $\boldsymbol{\varepsilon}_{\mathbf{Li}}$ is the lithiation/delithiation-induced strain, Ω is the partial molar volume of the Li atom, c_s is the current timestep Li concentration and $c_{s_{ini}}$ is the initial Li concentration in the NMC particle. The total strain is the linear sum of elastic strain and lithiation-induced strain.

$$\text{Constitutive relation:} \quad \boldsymbol{\sigma} = \mathbf{C}: \boldsymbol{\epsilon}_{\mathbf{el}} \qquad (5)$$

Where $\boldsymbol{\sigma}$ is the stress, $\mathbf{C}$ is the elastic stiffness matrix.

$$\text{Mechanical equilibrium:} \quad \nabla \cdot \boldsymbol{\sigma} = 0. \qquad (6)$$

The model is constrained using symmetric boundary conditions on both sides and fixed boundary conditions at the top and bottom. The diffusivity and reaction rate constant for the Butler Volmer equation follows the step function varying with the state of charge[1]. The interfacial reaction occurs at the boundary of the carbon binder, where it touches the NMC particle. The model is simulated for C/10 charging condition where the maximum capacity of NMC is defined as 49,000 mol/m^3. The domains inside NMC particle exhibit directionally isotropic mechanical (Elastic modulus = 140 GPa and Poisson ratio = 0.3) and transport properties. The carbon binder has high electrical conductivity (10^3 S/m), low elastic modulus = 5 GPa, and Poisson ratio = 0.3. As Li moves out of NMC domains, a volumetric change of 3% occurs, leading to stress at domain boundaries and causing damage. The stress in the domain boundary spring (σ_{spring}) is calculated using: $\sigma_{spring} = K_{spring}\delta_{spring}$, where K_{spring} is the spring stiffness (= 10^{15} N/m^2) and δ_{spring} is the deformation of spring. The damage (Dmg) is calculated as:

$$\text{Dmg} = \begin{cases} 0, & \delta_{spring} < \delta_0 \\ \frac{\delta_{spring}-\delta_0}{\delta_d-\delta_0}, & \delta_0 < \delta_{spring} < \delta_d \\ 1, & \delta_{spring} > \delta_d \end{cases} \qquad (7)$$

We use MUMPS (MUltifrontal Massively Parallel sparse direct Solver), a built-in time-dependent solver in COMSOL Multiphysics, to solve lithium concentration, stress, and domain boundary damage evolution.

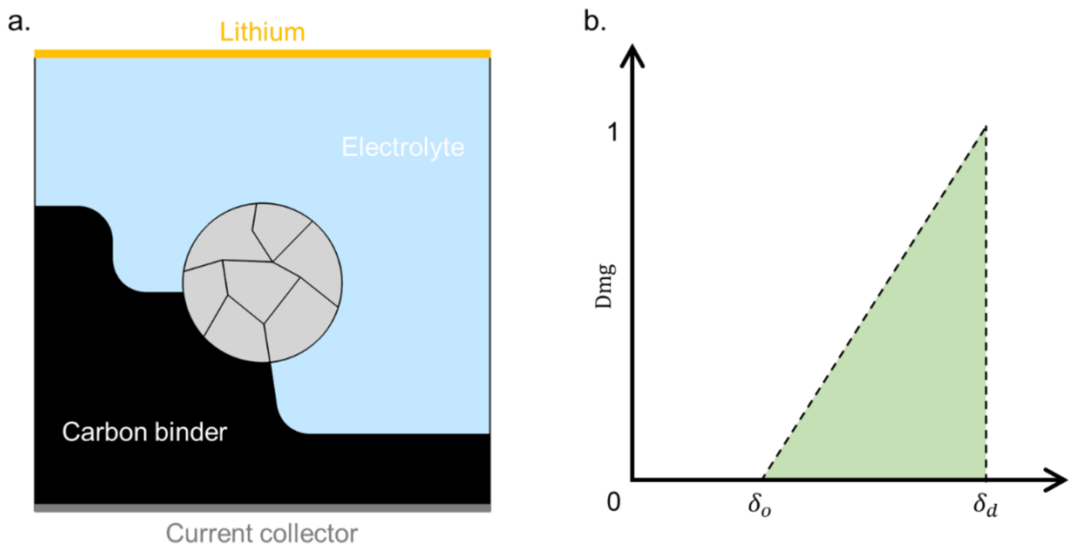

Figure S12 (**a**) Schematic of a half-cell geometry used in our finite element model. The current collector at the bottom connects the external circuit to the electrode's carbon binder (black color). An NMC particle (light grey color) is divided into domains and is surrounded by liquid electrolyte (light blue color). (**b**) The homogeneous domains inside NMC are connected by elastic springs at the boundary. As the spring extension reaches δ_o, the damage (Dmg) begins to grow linearly from the initial value of zero with further deformation. Once the Dmg reaches the value of 1 (spring extension = δ_d), the boundary is completely damaged, and Li transport across the domain boundary is reduced to zero.

Reference

1. Sharma N, Vasconcelos LSd, Hassan S, Zhao K. Asynchronous-to-Synchronous Transition of Li Reactions in Solid-Solution Cathodes. *Nano Letters* **22**, 5883-5890 (2022).

2. Li L, Xie Y, Maxey E, Harder R. Methods for operando coherent X-ray diffraction of battery materials at the Advanced Photon Source. *Journal of Synchrotron Radiation* **26**, 220-229 (2019).

3. Merryweather AJ, Schnedermann C, Jacquet Q, Grey CP, Rao A. Operando optical tracking of single-particle ion dynamics in batteries. *Nature* **594**, 522-528 (2021).

4. Xu C, *et al.* Operando visualization of kinetically induced lithium heterogeneities in single-particle layered Ni-rich cathodes. *Joule* **6**, 2535-2546 (2022).

5. Merryweather AJ, Jacquet Q, Emge SP, Schnedermann C, Rao A, Grey CP. Operando monitoring of single-particle kinetic state-of-charge heterogeneities and cracking in high-rate Li-ion anodes. *Nature Materials* **21**, 1306-1313 (2022).
