# Supplementary material for: Asynchronous domain dynamics and equilibration in layered oxide battery cathode
Source: Nat Commun. 2023 Dec 18;14:8394. doi: 10.1038/s41467-023-44222-x (PMC10728132; doi:10.1038/s41467-023-44222-x)
Supplement: Supplementary file 3 — Description of Additional Supplementary Files [file 41467_2023_44222_MOESM3_ESM.pdf]

Description of Additional Supplementary Files

Supplementary Movies

Supplementary Movie 1.
Operando CMCD patterns of the Debye–Scherrer ring over the (003) peak from an operating cell with NMC cathode.

Supplementary Movie 2.
Charing-induced evolution of lithium concentration and damage profiles from finite element analysis of an NMC particle comprising intra-particle domains.
